# Molecular docking analysis of α-Synuclein aggregation with Anle138b

**DOI:** 10.6026/973206300200217

**Published:** 2024-03-31

**Authors:** Annu Grewal, Deepak Sheokand, Vandana Saini, Ajit Kumar

**Affiliations:** 1 Toxicology and Computational Biology Group, Centre for Bioinformatics, Maharshi Dayanand University, Rohtak, Haryana, India

**Keywords:** Molecular docking, protein-protein docking, Parkinson's disease, α-synuclein aggregation

## Abstract

α-Synuclein aggregation into toxic oligomeric species is central to Parkinson's disease pathogenesis. Anle138b is a recently identified inhibitor of α-synuclein oligomerization showing promise in preclinical studies. This study employed computational approaches to elucidate Anle138b's mechanism of oligomer-specific action. The inhibitory potential of Anle138b against α-synuclein oligomers was evaluated by performing molecular docking studies using AutoDock Tools, followed by their binding pocket analysis. Further, protein-protein docking studies were performed using Hex8.0 to validate the aggregation inhibitory potential of Anle138b. Molecular docking revealed increasing binding affinity of Anle138b against higher order α-synuclein oligomers (dimer to decamer). Anle138b occupied oligomeric cavity and interacted with residues Thr54, Gly73, Val74 and Thr75 across several oligomers. Protein-protein docking showed that Anle138b interferes with α-synuclein decamer formation. These results highlight the oligomer-directed inhibitory mechanism of Anle138b, without hindering the monomeric forms and provide molecular insights to advance its therapeutic development for Parkinson's and related synucleinopathies.

## Background:

α-Synuclein oligomerization is considered a crucial event in the pathogenesis of Parkinson's disease (PD). α-Synuclein is a small, presynaptic protein whose physiological function is not entirely understood, but it is believed to play a role in neurotransmitter release and synaptic function [1]. However, under pathological conditions, α-synuclein can misfold and aggregate, forming oligomers, protofibrils, and eventually insoluble fibrils that accumulate in Lewy bodies, the pathological hallmark of PD and other synucleinopathies [2]. Several studies have highlighted the importance of α-synuclein oligomers, rather than the larger fibrillar aggregates, as the primary toxic species responsible for neurodegeneration in PD [3]. Oligomeric α-synuclein has been shown to disrupt cellular functions, induce oxidative stress, impair protein degradation pathways, and promote neuroinflammation [4]. Additionally, α-synuclein oligomers can interact with cellular membranes, leading to increased permeability and disruption of cellular homeostasis [5]. The process of α-synuclein oligomerization is thought to be a critical step in the disease cascade, as it precedes the formation of larger, insoluble aggregates [6]. Factors such as oxidative stress, post-translational modifications, and genetic mutations can influence the propensity of α-synuclein to oligomerize and aggregate [7]. Moreover, the oligomerization process can be self-propagating, as oligomers can seed the conversion of monomeric α-synuclein into additional oligomers, facilitating the spread of pathology throughout the brain [8]. Targeting α-synuclein oligomerization has emerged as a promising therapeutic strategy for PD and related synucleinopathies [9]. Several approaches have been explored, including the development of small molecules, antibodies, and peptide inhibitors that can interfere with the oligomerization process or promote the clearance of existing oligomers [10]. Additionally, modulating cellular pathways involved in protein quality control, such as the ubiquitin-proteasome system and autophagy, may help reduce the accumulation of toxic α-synuclein species [11]. In conclusion, the oligomerization of α-synuclein is widely recognized as a pivotal event in the pathogenesis of Parkinson's disease [12]. Targeting this process through various therapeutic approaches holds great promise for developing effective treatments that can slow or halt the progression of PD. To inhibit the α-synuclein aggregation various therapeutics are in preclinical trials, including molecular chaperons (Hsp70 and Hsp104), molecular tweezer (CRL01), prolyl oligopeptidase inhibitor (KYP-2047) and oligomer modulator (Anle138b), showing promising neuroprotection and decreased α-synuclein pathology [13]. Anle138b was first identified using high-throughput screening for small-molecule inhibitors of α-synuclein oligomerisation. In vitro studies demonstrated Anle138b's ability to inhibit the oligomerization of pathogenic proteins like prion protein (PrP) and α-synuclein while allowing the formation of less harmful amyloid fibrils. In vivo experiments using transgenic mouse models showed that Anle138b treatment reduced the accumulation of toxic oligomers, improved pathological phenotypes like motor impairments and neuronal loss, and suggested a favorable safety profile and bioavailability [14]. Therefore, it is of interest to investigate the possible mechanisms of action and potential interactions of Anle138b with various oligomeric conformations of α-synuclein.

## Methodology:

## Molecular docking studies:

Molecular docking experiments were undertaken in a stepwise, systematic manner to evaluate Anle138b's inhibitory activity against the in-house generated α-synuclein oligomeric conformations of dimer (I-I), trimer (II-I), tetramer (II-II), pentamer (IV-I), hexamer (V-I), heptamer (VI-I), octamer (IV-IV), nonamer (V-IV) and decamer (IV-VI) using AutoDock 4.2 [15]. All the forms of target receptor protein α-synuclein (monomer to decamer) and the ligand (Anle138b), were prepared for molecular docking studies using AutoDock Tools [16]. Polar hydrogens, Gasteiger and Kollman charges were added at each preparation (monomer to decamer) of the receptor protein α-synuclein. The various docking parameters used for the grid parameter file generation for the target receptor proteins (monomeric to decameric α-synuclein) are listed in Table 1. Molecular docking was performed using 100 runs of the Lamarckian genetic algorithm at a mutation rate of 0.02 and a crossover rate of 0.8, with a population size of 300. The output files (.dlg) of molecular docking studies of the target receptors (monomeric to decameric α-synuclein) with Anle138b were analysed for various binding poses and the binding poses with maximum cluster size and minimum binding energy were selected for further studies of their binding pocket interactions.

## Binding pocket studies:

The binding pocket interactions of the selected binding poses of Anle138b with α-synuclein oligomeric conformations of dimer (I-I), trimer (II-I), tetramer (II-II), pentamer (IV-I), hexamer (V-I), heptamer (VI-I), octamer (IV-IV), nonamer (V-IV) and decamer (IV-VI) were analysed using AutoDock Tools.

## Protein-protein docking studies:

The decameric form of α-synuclein oligomerization, being the primary stage of fibrilization [17], was selected for the validation studies of Anle138b as an aggregation inhibitor. Protein-protein docking studies were performed using Hex8.0 [18] in the presence and absence of Anle138b, using the default parameters of protein-protein docking i.e. Correlation type - Shape + Electro; FFT Mode - 3D; Grid dimension - 0.6; Step size - 7.5; Box size - 10; Sampling method - Range angles; Solutions - 2000; Receptor range - 180; Ligand range - 180; Twist range - 360; Distance range - 40; Steric scan - 18; and Final search - 25. Four combinations were made having respective binding energies as [(Hexamer+Anle138b)+(Tetramer+Anle138b)=B1], [(Hexamer+Anle138b)+ (Tetramer)=B2], [(Hexamer)+ (Tetramer+Anle138b)=B3], [(Hexamer)+ (Tetramer)= BN].

## Results and Discussion:

## Molecular docking studies:

The various combinations of the dimer (I-I), trimer (II-I), tetramer (II-II), pentamer (IV-I), hexamer (V-I), heptamer (VI-I), octamer (IV-IV), nonamer (V-IV) and decamer (IV-VI) forms of α-synuclein protein were docked against Anle138b, using AutoDock 4.2, to check its potential as an oligomerisation inhibitor. The binding energies and the inhibition constants for the docked complexes of monomeric to decameric α-synuclein protein were observed to vary in decreasing order, implying the increasing inhibitory potential of Anle138b as oligomerisation proceeds (Table 2). The binding energy was observed to decrease successively from monomer (-6.30 kcal/mol) to hexamer (-8.29 kcal/mol), also the inhibition constant value was observed to fall from 24.10 µM for monomeric α-synuclein-Anle138b docked complex, to 0.845 µM for hexameric α-synuclein-Anle138b docked complex. Slightly increased binding energy was observed for heptameric α-synuclein-Anle138b docked complex (-8.27 kcal/mol), in comparison to hexameric α-synuclein-Anle138b docked complex, and was observed to decrease successively after that, till nonameric α-synuclein-Anle138b docked complex (-8.78 kcal/mol). A similar pattern was observed of the inhibition constant, 0. 865 µM for heptameric α-synuclein-Anle138b docked complex and then falling till nonameric α-synuclein-Anle138b docked complex (0.368 µM). Overall, the binding energy of Anle138b with the α-synuclein oligomers was observed to decrease from -6.30kcal/mol (monomeric α-synuclein) to -8.38kcal/mol (decameric α-synuclein), also the inhibition constant value fall from 24.10 µM (monomeric α-synuclein) to 0.719 µM (decameric α-synuclein).

## Binding pocket studies:

**Figure 1:**
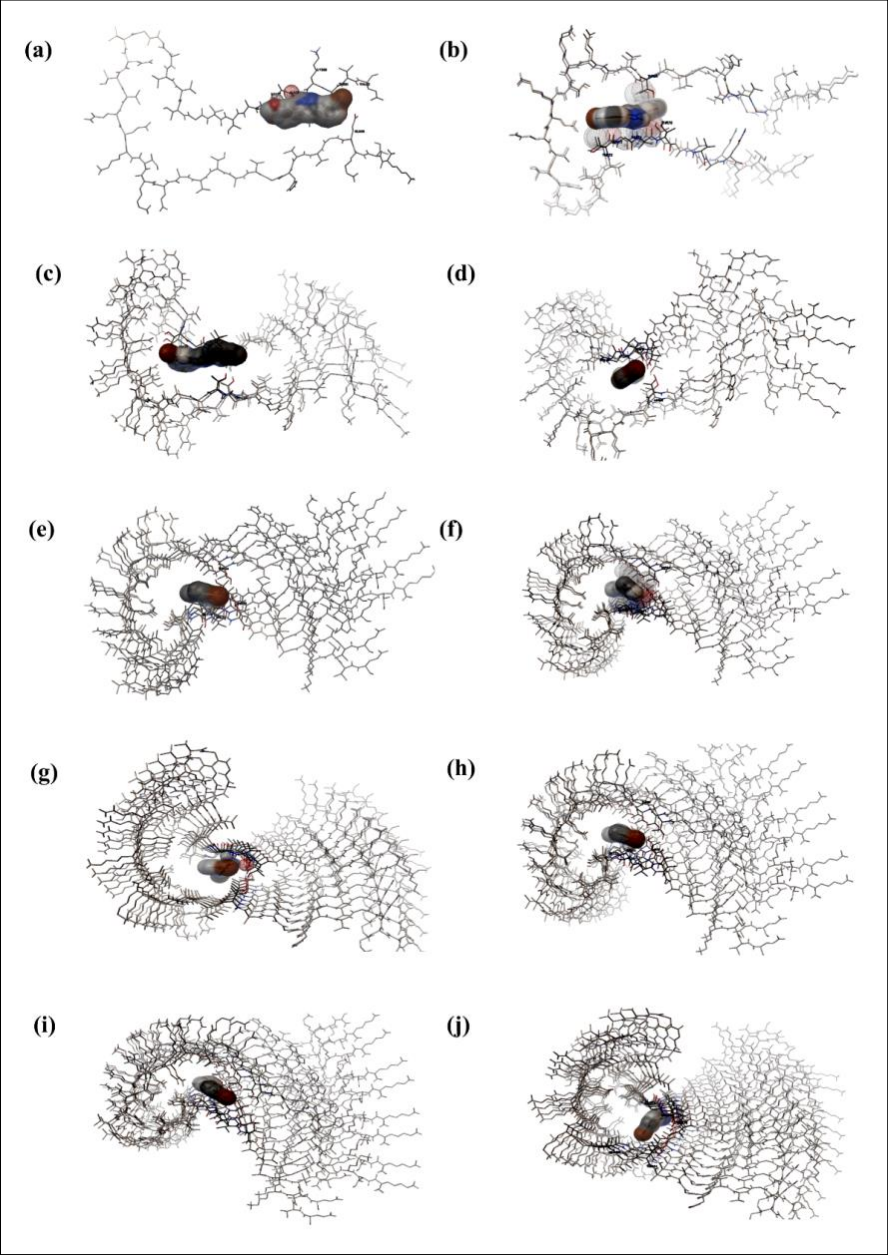
Binding of Anle138b in the cavity of α-synuclein oligomeric conformations (a) monomeric α-synuclein, (b) dimeric α-synuclein, (c) trimeric α-synuclein, (d) tetrameric α-synuclein, (e) pentameric α-synuclein, (f) hexameric α-synuclein, (g) heptameric α-synuclein, (h) octameric α-synuclein, (i) nonameric α-synuclein, (j) decameric α-synuclein.

**Figure 2:**
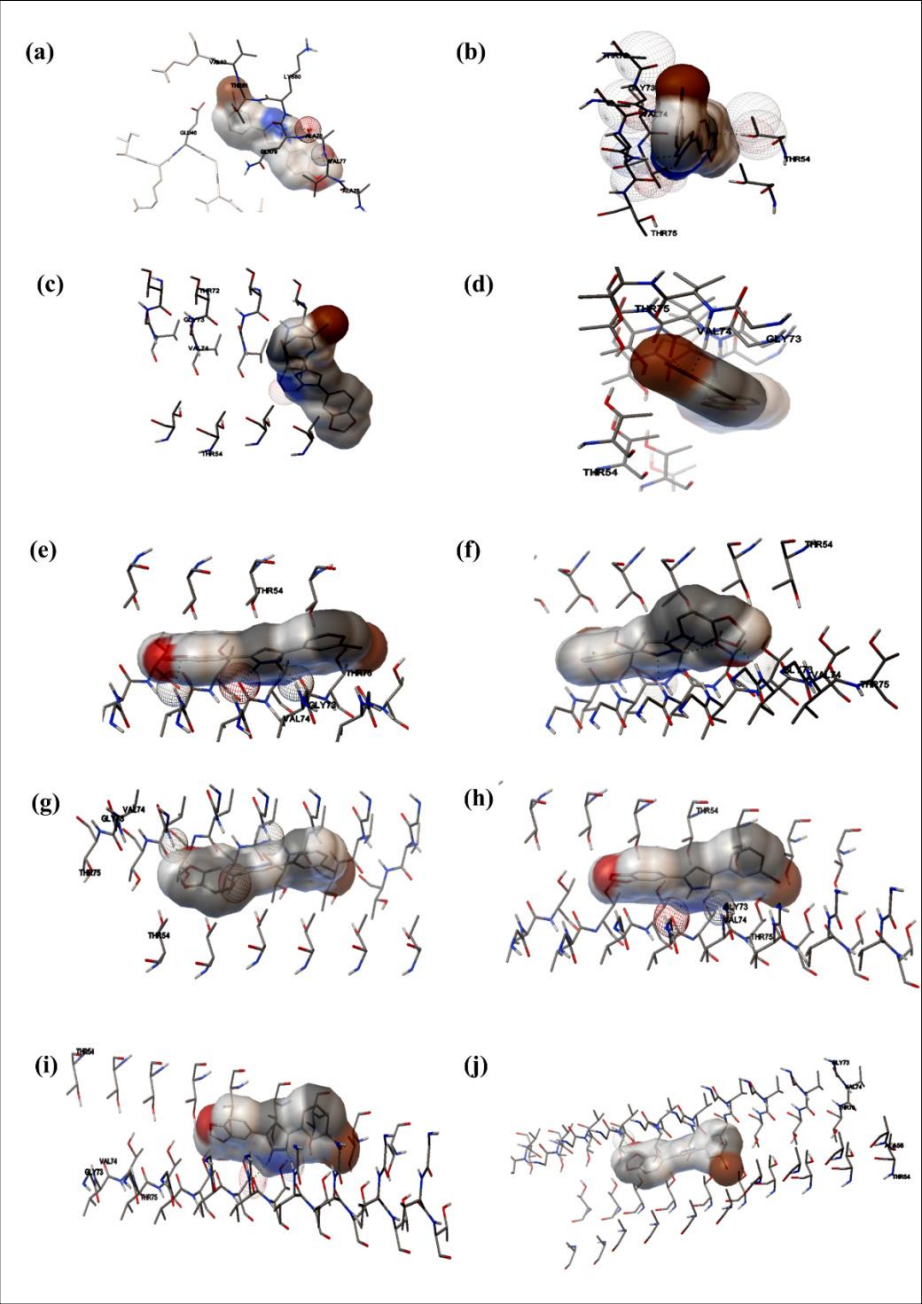
Interacting residues of the α-synuclein oligomeric conformations (a) monomeric α-synuclein, (b) dimeric α-synuclein, (c) trimeric α-synuclein, (d) tetrameric α-synuclein, (e) pentameric α-synuclein, (f) hexameric α-synuclein, (g) heptameric α-synuclein, (h) octameric α-synuclein, (i) nonameric α-synuclein, (j) decametric α-synuclein, docked against Anle138b.

The binding pockets of dimer (I-I), trimer (II-I), tetramer (II-II), pentamer (IV-I), hexamer (V-I), heptamer (VI-I), octamer (IV-IV), nonamer (V-IV) and decamer (IV-VI) forms of α-synuclein protein docked against Anle138b were analysed using AutoDockTools. Anle138b was observed to occupy the cavity of oligomeric conformations having multiple hydrophobic and hydrogen bond interactions (Figure 1a,Figure 1b,Figure 1c, Figure 1d,Figure 1e,Figure 1f, Figure 1g,Figure 1h,Figure 1i, Figure 1j, Table 2). Thr54, Gly73, Val74 and Thr75 were found to be the common residues of the α-synuclein oligomers (tetramer to decamer), interacting with Anle138b (Figure 2d,Figure 2e,Figure 2f, Figure 2g,Figure 2h,Figure 2i, Figure 2j). Hydrogen bonds were observed to vary between 2-3 from monomeric and decameric conformations of α-synuclein docked with Anle138b (Figure 2a,Figure 2b, Figure 2c,Figure 2j). A constant number of hydrogen bonds was observed from tetrameric to heptameric α-synuclein docked with Anle138b (Gly73, Val74 and Thr75). Gly73 and Val74 remained constant hydrogen-bond forming residues for octameric, nonameric and decameric forms of α-synuclein docked with Anle138b.

## Protein-protein docking studies:

Decamerization of α-synuclein was selected for the study, being the primary stage of fibrilization and the binding affinity for the creation of decameric (IV-VI) form having respective binding energies as [(Hexamer+Anle138b)+(Tetramer+Anle138b)=B1], [(Hexamer+Anle138b) +(Tetramer)=B2], [(Hexamer)+(Tetramer+Anle138b)=B3], [(Hexamer)+(Tetramer)=BN] were compared (Table 3). The binding energies of the α-synuclein oligomeric forms complexed with Anle138b followed a descending order as B1>B2>B3> BN, implying that the affinity of aggregation decreases when the α-synuclein oligomers are complexed with Anle138b.

## Conclusion:

We evaluated the inhibitory mechanism of the compound Anle138b against α-synuclein aggregation at various oligomeric stages, from dimer to decamer, using computational approaches. Molecular docking analyses revealed that Anle138b displayed increasing binding affinity and inhibition potential against higher order α-synuclein oligomers, with the most favourable binding observed against nonameric and decameric conformations. Anle138b was found to occupy the oligomeric cavity and interact with key hydrophobic and hydrogen bonding residues like Thr54, Gly73, Val74 and Thr75 across several α-synuclein oligomers. Protein-protein docking experiments also demonstrated that Anle138b could interfere with and decrease the affinity for α-synuclein decamer formation, which is a critical event preceding fibrillization. Taken together, these computational findings provide valuable insights into the mechanism of Anle138b as an oligomerization inhibitor - by directly binding to oligomeric intermediates with high affinity, blocking their aggregation into toxic, fibrillar end-products. The oligomer-specific action and increased inhibitory potency of Anle138b against later stage oligomers provide complementary in-silico support to its promising therapeutic potential for Parkinson's and other synucleinopathy diseases characterized by pathogenic α-synuclein aggregation.

## Figures and Tables

**Table 1:** Grid parameter files dimensions for the target protein receptors (Monomeric to decametric α-synuclein)

**α-Synuclein formations**	**Number of grid points in XYZ direction**	**Spacing(A)**	**Grid-center**
Monomer	158 104 40	0.375	8.734 10.633 -2.468
Dimer (I-I)	160 110 58	0.375	8.396 9.531 -5.529
Trimer (II-I)	170 120 64	0.375	8.524 12.063 -2.33
Tetramer (II-II)	178 116 68	0.375	11.155 13.856 3.392
Pentamer (IV-I)	176 124 86	0.375	10.599 13.645 0.302
Hexamer (V-I)	178 126 96	0.375	10.703 13.396 -2.782
Heptamer (VI-I)	194 132 104	0.375	11.275 13.144 -4.489
Octamer (IV-IV)	192 142 110	0.375	12.59 15.002 11.155
Nonamer (V-IV)	126 126 126	0.375	8.994 15.418 9.356
Decamer (IV-VI)	180 122 126	0.375	12.359 16.043 7.408

**Table 2:** Molecular docking studies of the α-synuclein oligomeric conformations (Monomer to decamer) against Anle138b

**α-synuclein formations**	**Binding energy (kcal/mol)**	**Inhibition constant (Ki in µM)**	**Nearby interacting residues**	**Hydrogen bond-forming residues**
Monomer	-6.3	24.1	Glu46, Ala76, Val77, Ala78, Gln79, Lys80, Thr81, Val82	Val77, Ala78
Dimer (I-I)	-6.34	22.69	Thr54, Thr72, Gly73, Val74, Thr75	Val74, Thr75
Trimer (II-I)	-7.32	4.33	Thr54, Thr72, Gly73, Val74	Val74, Gly73
Tetramer (II-II)	-7.97	1.45	Thr54, Gly73, Val74, Thr75	Gly73, Val74, Thr75
Pentamer (IV-I)	-8.21	0.956	Thr54, Gly73, Val74, Thr75	Gly73, Val74, Thr75
Hexamer (V-I)	-8.29	0.845	Thr54, Gly73, Val74, Thr75	Gly73, Val74, Thr75
Heptamer (VI-I)	-8.27	0. 865	Thr54, Gly73, Val74, Thr75	Gly73, Val74, Thr75
Octamer (IV-IV)	-8.72	0.402	Thr54, Gly73, Val74, Thr75	Gly73, Val74
Nonamer (V-IV)	-8.78	0.368	Thr54, Gly73, Val74, Thr75	Gly73, Val74
Decamer (IV-VI)	-8.38	0.719	Thr54, Ala56, Gly73, Val74, Thr75	Gly73, Val74

**Table 3:** Binding energies of the α-synuclein oligomeric forms complexed with Anle138b

**S. No.**	**Complex**	**Binding energy (kJ/mol)**
1	(Hexamer+Anle138b)+(Tetramer+Anle138b)	-545.68
2	(Hexamer+Anle138b)+(Tetramer)	-813.79
3	(Hexamer)+(Tetramer+Anle138b)	-931.04
4	(Hexamer)+(Tetramer)	-1038.42
